# DAergic Neuronal Dynamics: intrinsic properties, receptor dynamics, and network effects

**DOI:** 10.1186/1471-2202-14-S1-P156

**Published:** 2013-07-08

**Authors:** A Oster, B Gutkin

**Affiliations:** 1 Department of Mathematics, Case Western Reserve University, Cleveland, OH 44016, USA; 2 Group for Neural Theory, Ecole Normale Supérieure, Paris, 75005, France; 3 Centre National de la Recherche Scientifique, Paris, France

## 

Midbrain dopaminergic neurons send numerous projections to cortical and sub-cortical areas, and, in a manner dependent upon their activities, diffusely release dopamine (DA) to their targets. In particular, in the ventral tegmental area (VTA), the release of dopamine is thought to be associated with reward-related information [1]. The DAergic neurons display a wide range of activity modes varying in frequency and spike distribution, with some cells predominately firing in a tonic fashion while others exhibit burst firing [2]. The variety of activity modes is significant as experimental studies have shown that DAergic neuronal bursting is associated with a greater degree of DA release than an equivalent tonic activity pattern [3]. Here, we introduce a single compartmental, conductance-based computational model for DA cell activity and utilize it to identify mechanisms behind certain activity modes displayed by midbrain DA neurons (in line with experimental findings and other modeling studies). Our model suggests that endogenous burst firing patterns are strongly dependent upon the strength of the SK conductance, the amount of drive, and the amount of GABA inhibition. The model has the property that transient burst firing events can be elicited by an increase in the NMDA conductance, and by sudden decreases of the GABA inhibition. In addition to these findings, we present our current progress on the development of a meta-model for DA cell activity that considers a merging of our conductance-based model with detailed receptor dynamics, mean-field circuit dynamics, and variations in the cortical drive from the prefrontal cortex. We, also, explore how nicotine stimulation leads to an increase in the firing rate and, for certain neurons, an increase in the degree of burst firing.
